# When Technology Falters: Ensuring Donor Safety and System Reliability Amid Apheresis Technical Challenges

**DOI:** 10.1111/aor.70097

**Published:** 2026-01-14

**Authors:** Bhagyashri Surve, Chitra Dewanji, Aditi Khanna, Suryatapa Saha, Shashank Ojha

**Affiliations:** ^1^ Tata Memorial Centre Advanced Centre for Treatment, Research & Education in Cancer, HBNI Navi Mumbai India

**Keywords:** donor retention, Ishikawa diagram, oncology center, plateletpheresis, root cause analysis, technical malfunction, the 5 “whys” technique

## Abstract

With the rising demand for platelet products, the use of apheresis‐derived single donor platelets (SDPs) has grown substantially. While plateletpheresis is generally considered safe, it is not devoid of complications. This retrospective descriptive study reviews three adverse events that occurred during SDP collection at a transfusion medicine department. Root cause analysis (RCA) was employed using tools such as the 5 “Whys” technique and the Ishikawa (Fishbone) Diagram to identify underlying causes and propose preventive strategies. In the first incident, a first‐time donor experienced vasovagal symptoms triggered by a visible blood leak from the apheresis kit. The second incident involved a centrifuge alarm and mid‐procedure halt due to blood spillage caused by improper kit installation. The third incident was identified post‐procedure when the platelet concentrate was found contaminated with red blood cells from a manufacturing defect. All donors recovered without lasting health effects; however, such technical or procedural failures can erode donor confidence, reduce willingness for repeat donations, and impact retention of valuable apheresis donors. The events in this case series highlight that adverse outcomes can stem from both equipment‐related faults and human errors. Strengthening the voluntary apheresis donor base in India requires prioritizing donor safety, ensuring positive donation experiences, comprehensive staff training, adherence to standard operating procedures (SOPs), and fostering a supportive, trust‐based environment.

## Graphical abstract

This study reviews three adverse events during single donor platelet collection using root cause analysis. Causes included human error, equipment defects, and procedural lapses. Though all donors fully recovered, the incidents underscore the need for strict SOP adherence, staff training, and donor safety to maintain trust and donation retention. The figure shows the fishbone diagram explaining the different causes that can lead to poor quality collection of single donor platelets.

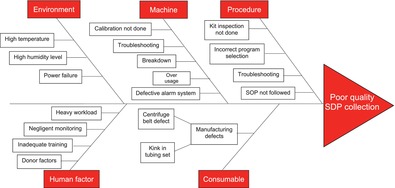

## 1 Background

Platelet transfusion is a critical supportive therapy in oncology, particularly for preventing bleeding in thrombocytopenic patients. In a plateletpheresis procedure, also known as single donor platelet (SDP) collection, the donor is connected to an apheresis machine which removes the donor's platelets and some plasma from their whole blood, and the rest of the components are returned to the donor [1]. The rising demand for platelet transfusions has led to increased use of apheresis‐derived single donor platelets, which, while efficient, carry both common and unique complications due to the collection method and donation frequency [2].

Over the years, several apheresis platforms have been utilized. Blood centers always seek suitable systems that have stringent safety systems along with the versatility to perform as many procedures as possible. Computer innovations have made it possible with current apheresis technology to obtain more real‐time information of the procedure, which permits the operator to adjust parameters not only to optimize the specific procedure but also to safeguard against potential adverse events [3].

Although apheresis procedures are generally considered safe, they are not entirely free from risk. While technologically advanced, they are still susceptible to machine or human‐related errors. Adverse events, though often minor, can still occur and not only compromise product integrity but may impact donor comfort and willingness to return for future donations. Their occurrence can be significantly reduced through vigilant monitoring of donors before, during, and after the procedure. Additionally, implementing robust training programs for technical personnel and ensuring the continuous supervision of experienced transfusion medicine specialists play a crucial role in maintaining donor safety and improving outcomes [4].

Root Cause Analysis (RCA) helps identify underlying issues after adverse events, enabling effective, preventive strategies. By mastering RCA, healthcare professionals can improve patient safety, drive meaningful change, and fulfill mandated institutional responsibilities to reduce errors and enhance care quality [5]. This case series presents three such events, highlighting the value of root cause analysis in identifying failures and improving system processes.

## 2 Aim and Objectives

**Table:** 

Primary objective	To examine failure incidents in the final stages of single donor plateletpheresis and identify the root causes of each malfunction.
Secondary objective	Assess impact on donor safety and product quality and evaluate the preventability of each.

## 3 Material and Methods

This is a retrospective descriptive study which was conducted in the Department of Transfusion Medicine from January 2024 to December 2024 in a monocentric blood center. A total of 2154 SDP collection procedures were performed in 2024, of which the three unique incidents were taken. Written informed consent was obtained from all donors prior to initiating the SDP collection procedure as per departmental protocol. Details on malfunctions like detailed donor records, machine logs, and staff notes addressing the issue were retrieved from incident report in the departmental records. It also described the specific actions that were taken to rectify the situation in accordance with departmental protocol.

The data was analyzed to include multiple incidents. A detailed root cause analysis was performed using tools like the Fishbone Diagram and 5 “Whys”. This evaluation was performed to identify the important effects on donor safety, product quality, and procedural efficiency.

## 4 Case Details

### 4.1 Incident 1

**FIGURE 1:**
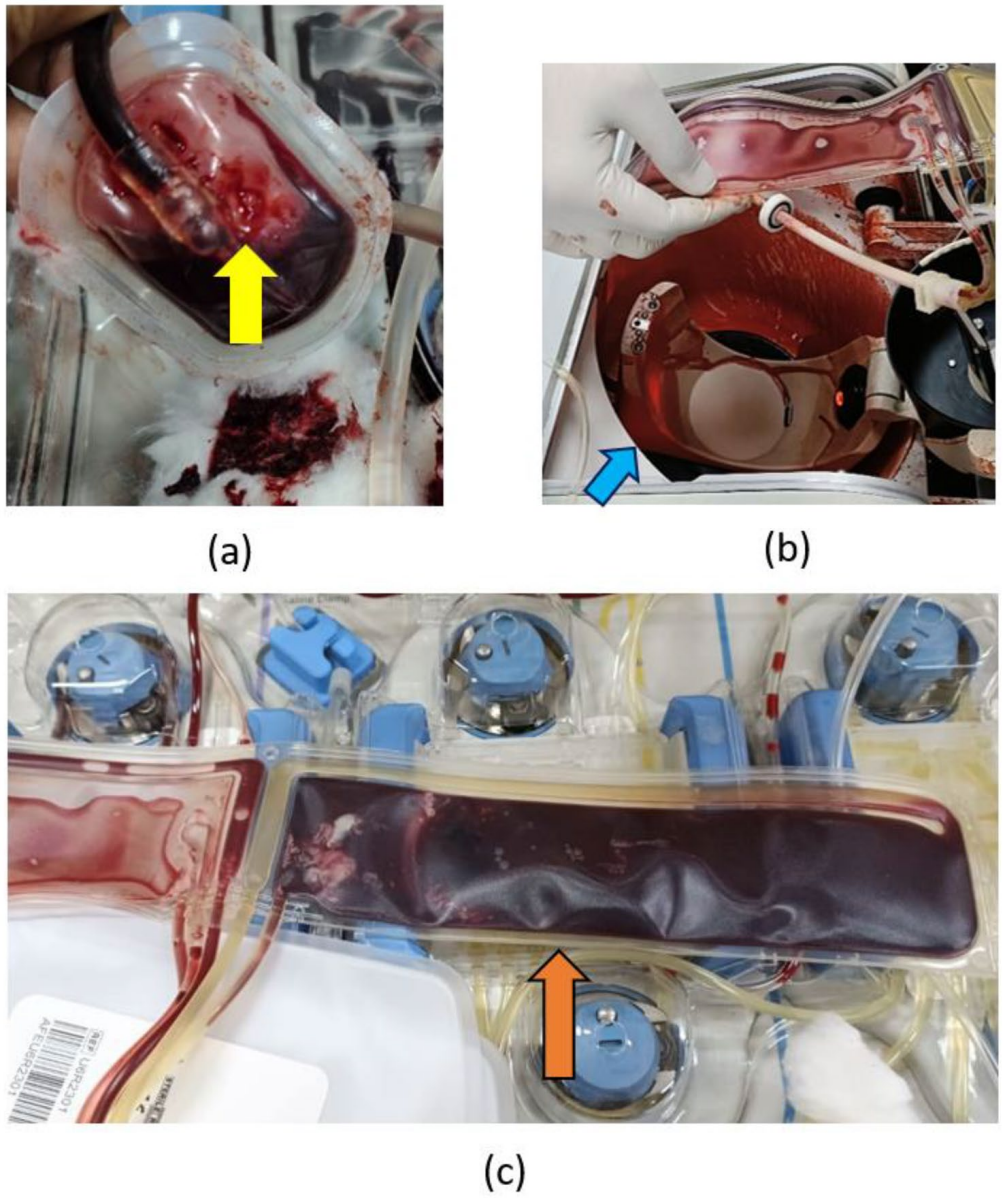
Images of each incident. (a) Small hole seen in the tubing in Incident 1. (b) Blood spillage seen in centrifuge chamber in Incident 2. (c) Red blood cell contamination seen in platelet concentrate in centrifuge belt in Incident 3. [Color figure can be viewed at wileyonlinelibrary.com]

A first‐time donor was screened and cleared for SDP collection. He was connected to the cell separator and the procedure was progressing uneventfully. The reinfusion stage was initiated. After 5 min of that, leakage of blood from the apheresis kit was noticed. The donor then started experiencing vaso‐vagal‐like symptoms of dizziness and tingling sensation over his face. He was immediately disconnected from the cell separator and positioned in the Trendelenburg position. He was started on IV calcium gluconate 20 cc in 100 mL normal saline over 1 h. This approach, combined with engaging conversation, helped alleviate the symptoms. As the dizziness reduced, he was brought down to a lying down position. Refreshments were provided and the donor was observed for an hour before release. When the apheresis kit was inspected, a small hole in the tubing was observed as shown in Figure 1a. As the procedure was almost completed, the product was fully recovered. The donor was eligible for donation after 3 months as reinfusion was not completed; however, he did not return for another donation [1].

### 4.2 Incident 2

A repeat, regular donor was connected to the cell separator and the procedure was progressing uneventfully. At 60% completion, an abnormal sound was heard from the cell separator (specifically centrifuge chamber). Immediately after a “Centrifuge leakage detected” alarm was detected and the system came to a halt. Upon further inspection of the centrifuge chamber, a breakage of the apheresis kit was seen with spillage of blood in the centrifuge chamber as shown in Figure 1b. The donor was immediately disconnected and counseled about the situation. As the procedure was not completed, only half a unit of SDP was recovered. The donor, being a regular platelet donor, was upset about the outcome. He was counseled further to reassure him that he would be able to donate again down the line after a gap of 3 months as reinfusion was not performed this time [1]. The donor returned for repeat donation after 3 months.

### 4.3 Incident 3

A regular, repeat donor was connected to the cell separator and the procedure progressed uneventfully. After the completion of the procedure, the donor was disconnected and the process for reconstitution of the SDP was initiated. At this stage, the centrifuge chamber is opened to remove the centrifuge belt, and the plasma and platelet concentrate are mixed to reconstitute the SDP product. When the centrifuge belt was removed, it was observed that the platelet concentrate was entirely red blood cell (RBC) contaminated, as shown in Figure 1c. Hence, reconstitution was not possible due to the contamination. The entire product was discarded. The donor was reassured that the procedure was successful; however, due to technical reasons, the product was not suitable for issue. He was reassured that this could happen due to technical malfunction and was motivated to return for repeat donation. The donor came back for a repeat successful donation 15 days later, which was then uneventfully.

The deferral period mentioned in each case is in accordance with the guidelines set by the National Blood Transfusion Council (NBTC), India [1] and our departmental SOPs.

The impact on the donors was analyzed in each incident along with its preventability:

- In *Incident 1*, due to the unpleasant first experience, he was unwilling to come back for donation. This signifies how a negative experience can dissuade a donor from revisiting the possibility of donating in the future. However, due to technical issues, the level of preventability was low.
- In *Incident 2*, due to kit breakage, reinfusion was not completely performed. Hence, the donor was deferred for 3 months according to institutional guidelines. As the donor was a regular, repeat donor, despite being frustrated by the outcome, he returned for platelet donation after 3 months. This was possible due to adequate pre‐counseling information given to the donor at the previous donations. The preventability in this case was high as this was mainly due to inadequate staff training.
- In *Incident 3*, loss of product due to RBC contamination was discovered which was later concluded to be due to a technical malfunction. The donor, being a regular and repeat donor, was sufficiently motivated to donate platelets successfully again after 15 days. Here the preventability was low.

## 5 Discussion

The rising demand for blood transfusions amid a shrinking donor pool has driven increased use of automated blood collections, which, while efficient, present both common and unique complications related to their methods and donation frequency [6]. These unique complications, like machine and kit related issues, can lead to demotivate donors for SDP donation as they are unfamiliar with such effects as seen in an incident discussed above. Reducing medical errors requires system‐level interventions, not individual blame.

A study conducted by Bassi et al. [2] and an audit conducted by Dogra et al. [4], both concluded that while apheresis is largely safe, adverse events can occur and impact donor retention. Building a strong voluntary apheresis donor pool in India relies on comprehensive staff training, effective donor education, and, most importantly, creating a positive and comfortable donation experience to encourage continued donor participation as demonstrated in the cases discussed here.

All the incidents were evaluated to identify the root cause using two methods:

1. 5 “Whys” techniques
2. Fishbone/Ishihara diagram

**FIGURE 2:**
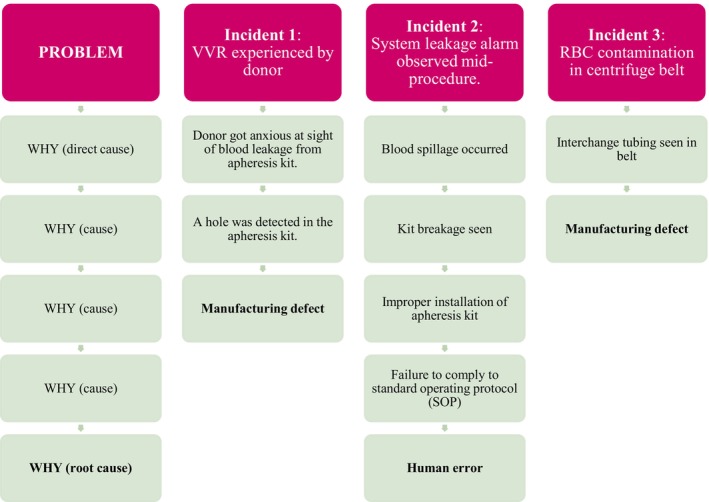
Flowchart of the 5 “Whys” technique (An adaptation of the study by Serrat [7]). [Color figure can be viewed at wileyonlinelibrary.com]

Serrat [7] proposed a five whys technique to explore cause‐and‐effect relationships. By asking “Why” five times, one can usually peel away the layers of symptoms that hide the cause of a problem. Five is a useful guideline, but depending on the situation, you may find that asking “Why” fewer or more times is necessary to reach the root cause. In this study, the technique was employed to achieve meaningful and well‐founded outcomes as shown in Figure 2.

**FIGURE 3:**
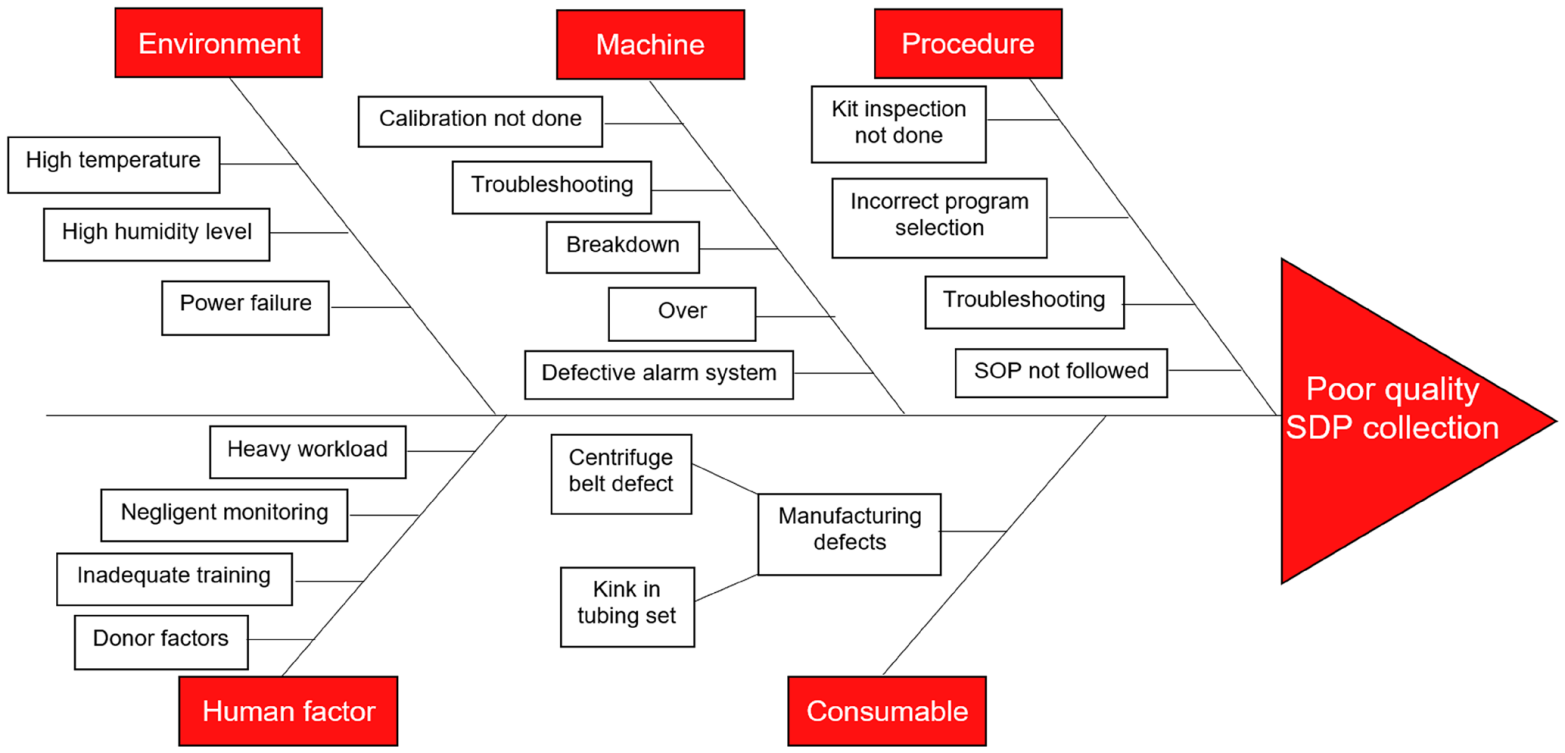
The fishbone diagram explaining the different causes that can lead to poor quality collection of single donor platelets. [Color figure can be viewed at wileyonlinelibrary.com]

Figure 2 above depicts how the root cause of each incident was deduced from the problem showcasing the cause‐and‐effect relationship. A fishbone diagram, also known as an Ishikawa or cause‐and‐effect diagram, is a visual tool used in root cause analysis to identify the reasons behind defects, variations, or failures in a process. Shaped like a fish skeleton, the diagram places the main problem at the “head” and maps out major causes (depicted by the red boxes) and their contributing root causes along the “bones” branching to the left. The structured approach, as shown in Figure 3, allows for a layered breakdown of potential factors, making it easier to systematically analyze and address the underlying issues [8].

In Figure 3 above, various factors affecting the quality of SDP collection are depicted in an Ishikawa diagram. Retaining donors holds higher value over recruiting new donors due to the following reasons [9]:

1. Risk of transfusion transmitted infections (TTIs) is higher in new recruits as they have not been priorly tested and hence have had a longer time to acquire infection than retained donors.
2. The mandatory screening procedure is longer and more time‐consuming for new recruits than for retained donors.
3. Donors have a healthier lifestyle than non‐donors as they are more motivated to donate.

The authors observed that nurturing a positive and reassuring donation experience remains one of the most influential factors in developing sustained donor commitment and repeat participation. Conversely, any adverse reaction—whether physical, technical, or emotional—can leave a lasting negative impression, creating hesitation or outright reluctance to donate again. A study by France et al. [10] demonstrated that even mild reactions, such as fainting, can discourage future donations. It was also noted that when such reactions are linked to technical issues, particularly during apheresis procedures, the impact on donors' trust can be consequential. As the apheresis donation is inherently longer and more technically involved than whole blood donation and donor is directly connected to cell separator machinery for an extended period. Therefore it was marked that any mechanical fault, alarm, or visible mishap can heighten donors' anxiety. Even if no physical harm occurs, the perception of risk can be enough to dissuade a donor from returning. This observation was particularly concerning when the affected donor is otherwise a strong candidate for regular platelet donation—losing such individuals represents a significant setback for the blood supply chain.

Since complete control over technical malfunctions is not always possible, the authors deduced that proactive strategies are essential like:

1. Use of comprehensive educational material along with one‐on‐one interaction with staff:
  It can help set realistic expectations, provide reassurance, and build trust so that donors remain motivated even if minor issues arise.
2. Staff training:
  Rigorous, ongoing staff training is pivotal—not only in equipment handling to minimize errors, but also in crisis management. For instance, when unexpected incidents occur, it was learnt that the staff responding in a calm and composed manner was able to comfort the donor and regain their confidence.

Every interaction leaves an imprint. As experienced by the authors, appropriate counseling along with meticulous staff education on equipment handling and crisis management has a positive impact on donor retention.

## 6 Conclusion

In conclusion, while automated blood collections help meet increasing transfusion demands, they also bring challenges such as machine or kit‐related issues that may affect donor comfort and retention. Despite being generally safe, apheresis‐related adverse events highlight the need for trained staff, informed donors, and a supportive donation environment. By combining technical excellence with empathetic communication, blood centers can transform potentially negative experiences into opportunities to strengthen donor relationships, ensuring their willingness to return and contribute again. These efforts are essential to developing and sustaining a strong voluntary apheresis donor pool.

## Author Contributions

B.S. and A.K. designed research, performed research, analyzed data, and wrote the manuscript; C.D. designed research, performed research and analyzed data and S.S. and S.O. designed research, supervised research, and edited the manuscript.

## Funding

The authors have nothing to report.

## Conflicts of Interest

The authors declare no conflicts of interest.

## Data Availability

The data that support the findings of this study are available from the corresponding author upon reasonable request.
